# Multilevel modeling and value of information in clinical trial decision support

**DOI:** 10.1186/s12918-014-0140-0

**Published:** 2014-12-24

**Authors:** Yuanyuan Cui, Brendan Murphy, Anastasia Gentilcore, Yugal Sharma, Lori M Minasian, Barnett S Kramer, Paul M Coates, John K Gohagan, Juergen Klenk, Bruce Tidor

**Affiliations:** Computer Science and Artificial Intelligence Laboratory and Program in Computational and Systems Biology, Massachusetts Institute of Technology, Cambridge, MA 02139 USA; Booz Allen Hamilton, Rockville, MD 20852 USA; Division of Cancer Prevention, National Cancer Institute, National Institutes of Health, Bethesda, MD 20892 USA; Office of Dietary Supplements, National Institutes of Health, Bethesda, MD 20892 USA; Office of Disease Prevention, Office of the Director, National Institutes of Health, Rockville, MD 20892 USA

**Keywords:** Multilevel modeling, Clinical trials, Decision support, Value of information (VOI), Cancer chemoprevention

## Abstract

**Background:**

Clinical trials are the main method for evaluating safety and efficacy of medical interventions and have produced many advances in improving human health. The Women’s Health Initiative overturned a half-century of harmful practice in hormone therapy, the National Lung Screening Trial identified the first successful lung cancer screening tool and the Prostate, Lung, Colorectal and Ovarian Cancer Screening Trial overturned decades-long assumptions. While some trials identify unforeseen safety issues or harms, many fail to demonstrate efficacy. Large trials require substantial resources; to ensure reliable outcomes, we must seek ways to improve the predictive information used as the basis of trials.

**Results:**

Here we demonstrate a modeling framework for linking knowledge of underlying biological mechanism to evaluate the expectation of trial outcomes. Key features include the ability to propagate uncertainty in biological mechanism to uncertainty in trial outcome and mechanisms for identifying knowledge gaps most responsible for unexpected outcomes. The framework was used to model the effect of selenium supplementation for prostate cancer prevention and parallels the Selenium and Vitamin E Cancer Prevention Trial that showed no efficacy despite suggestive data from secondary endpoints in the Nutritional Prevention of Cancer trial and found increased incidence of high-grade prostate cancer in certain subgroups.

**Conclusion:**

Using machine learning methods, we identified the parameters of the model that are most predictive of trial outcome and found that the top four are directly related to the rates of reactions producing methylselenol and transporting extracellular selenium into the cell as selenide. This modeling process demonstrates how the approach can be used in advance of a large clinical trial to identify the best targets for conducting further research to reduce the uncertainty in the trial outcome.

**Electronic supplementary material:**

The online version of this article (doi:10.1186/s12918-014-0140-0) contains supplementary material, which is available to authorized users.

## Background

When planning a cancer chemoprevention trial, researchers consider a large body of information to make critical decisions such as observational evidence, pre-clinical mechanistic understanding, pharmacology, and the risk and health of the study population. However, there is always uncertainty about efficacy of the intervention. Typically, investigators rely on results from previous trials or observational studies and use statistical modeling of disease incidence and other factors, such as participant compliance and attrition, to design a protocol with power sufficient to test the trial hypothesis [1,2]. They may consider evidence from mechanistic studies but the predictive value for clinical trials may be limited or unknown. While there is often biologic plausibility derived from the basic mechanisms that underlie the proposed intervention, the primary decision-making information is clinical. Identifying a means to integrate biologic information with clinical information and computational modeling may facilitate more informed decision making regarding trial design or whether to launch a large trial at all. Computational systems biology can directly model the biological mechanisms underlying trial interventions and inform the probabilistic assessment of trial outcomes. Mechanistic modeling can complement statistical approaches to trial planning and bridge the gap between clinical and basic science.

Developing mechanistic models of trial interventions is challenging because it relies on information of varied types, *in vitro* and *in vivo*, and from multiple levels: molecular, cellular, and tissue-level systems. On the other hand, clinical trial outcomes are measured at the trial participant level. Studying the effect of biologically-based therapeutic, diagnostic, or preventive interventions on cancer incidence in a clinical trial population requires a multilevel approach. Although there is growing interest in capturing the meaning of experimental and clinical data in mathematical models at each scale, few models have been developed that integrate across scales.

Another challenge is that there is often considerable uncertainty about the underlying biology involved in a clinical trial. Trial planners need the ability to estimate whether sufficient information exists to make decisions about trial design, identify critical gaps in knowledge and their potential effect on trial outcomes. This decision theoretic concept is commonly labeled Value of Information (VOI) analysis. Herein, we demonstrate an approach to VOI analysis, in which uncertainty about the biology of carcinogenesis and a proposed preventive intervention is propagated computationally across multiple scales to predict their influence on trial outcomes. VOI analysis is based on the evaluation of the probability of different outcomes for decisions made using uncertain information [3]. In trial planning it can be used to develop prospective estimates of the value of supplemental, pretrial data or to choose among study designs [4].

Integrating computational systems biology modeling into pre-trial decisions regarding agent readiness and trial design could bring added value by facilitating predictions of intervention effect or identifying gaps in knowledge. Here we demonstrate that a linked set of mathematical models can be used to simulate the connection between mechanistic biology and cancer incidence and to simulate a statistical sample of clinical trials in which supplements that affect the mechanistic biology are monitored to project the effect on cancer incidence. One valuable outcome of this approach is that uncertainties, gaps in knowledge, and underlying assumptions are made more explicit. A second advantage of the approach is the ability to identify which knowledge gaps dominate the uncertainty in trial outcome. Thus, mechanistic modeling can provide a valuable complement to the statistical models typically used in the planning and evaluation of clinical trials by allowing clinical trial decision makers to link models of the underlying biology of a trial to the trial outcomes to prioritize critical information needs. Here we illustrate how systems biology can be applied to clinical trial planning and also discuss the challenges of doing so.

## Methods

We formulate and link models from systems biology to models of cancer onset and natural history. Our case study was the Selenium and Vitamin E Cancer Prevention Trial (SELECT). Selenium was proposed as a promising chemopreventive agent [5,6], possibly through its role as an antioxidant and cofactor of enzymes such as glutathione peroxidase, but SELECT disappointingly found a non-statistically significant increase in prostate cancer incidence with selenium supplementation [7]. Further SELECT analysis found that selenium supplementation increased the risk of high-grade prostate cancer among men with high baseline toenail selenium status and that vitamin E increased the risk of prostate cancer among men with low toenail selenium status [8].

**Figure 1:**
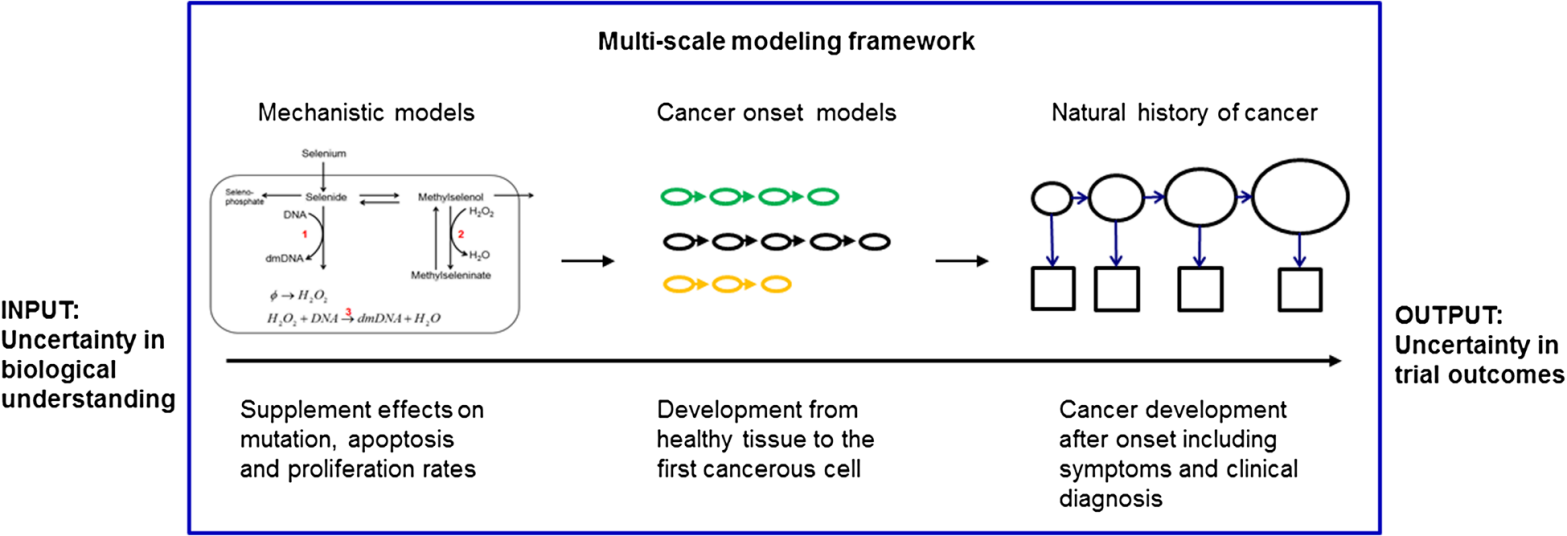
**Multilevel modeling framework.** Modeling across multiple levels connects biological level phenomena to population level outcomes. The lowest level describes the effects of trial interventions on metabolic and signaling pathways. Models at this scale determine the parameters of the tissue level cancer onset models, which describe malignant transformation. Finally, cancer onset models provide input to natural history models, which describe tumor progression, symptoms, and diagnosis. A full description of the biochemical and cancer onset model used here is given in Figure 2.

We use a multilevel modeling approach to quantitatively link uncertainty in biological mechanisms to uncertainty in trial outcome (Figure 1). Each of the three levels of modeling is developed independently and then linked. The biochemical model simulates selenium metabolism and its effects on DNA damage and repair, the cancer onset model simulates the stochastic accumulation of mutations leading to malignant transformation, and the natural history model is a waiting-time model representing an average lag time between cancer onset and clinical diagnosis. Each level of the model and the linkage mechanisms between them can be enhanced with added detail and modified to reflect accumulating scientific knowledge [9,10]. The modular approach allows for the testing of multiple models at each level to ensure robustness of results.

### Selenium metabolism model

**Figure 2:**
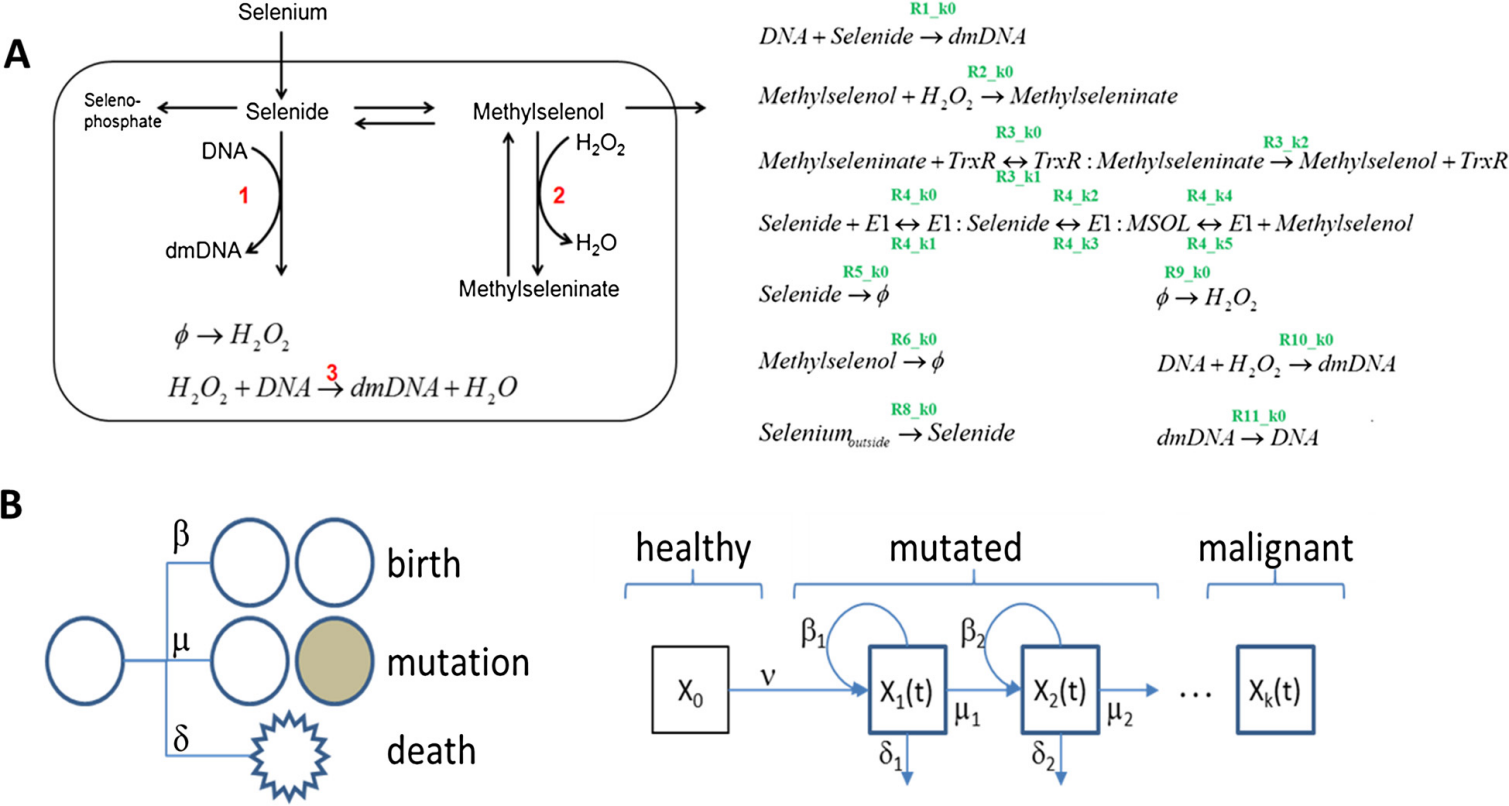
**Model descriptions. (A)**. A simplified model of selenium metabolism and the effects of its metabolites on DNA damage. The level of selenium affects the flux through reactions 1, 2, and 3, and can lead to low amounts of damaged DNA (dmDNA) when peroxide reduction (reaction 2) dominates or higher amounts when direct selenide damage to DNA (reaction 1) or peroxide-induced damage (reaction 3) dominates. A full list of reactions and model parameters is shown on the right. **(B)**. A multistage model represents cancer initiation as the progressive accumulation of mutations. Cells can divide symmetrically at rate β, divide with mutation at rate μ, or die at rate δ. A population of cells X_k_(t) with k mutations propagates through the division, death, and mutation of individual cells. When a cell mutates, it leaves the k-mutant population and enters the (k + 1)-mutant population. The initial population of healthy cells X_0_ produces mutated cells at a fixed rate ν and a cell is considered the first tumor cell when it progresses to a set number of mutations, often set at four, but can also be fit.

We developed an 18-parameter ordinary differential equation (ODE) model of a simplified version of selenium metabolism that describes time varying concentrations of chemical species and is parameterized by reaction rates (Figure 2A). The model is focused on the trade-off between protective effects of selenium, through methylselenol, and the DNA damaging effects of selenide [11-14]. The model allows us to study how selenium levels affect the rate of DNA damage.

### Cancer onset model

We implemented a 5-parameter cancer initiation model developed from existing models that describe the malignant transformation of healthy tissue through a series of stages defined by the accumulation of mutations, and terminating with the development of the first cancerous cell [15-17] (Figure 2B). Two competing concepts of carcinogenesis are somatic mutation theory (SMT) and tissue organization field theory (TOFT) [18,19]. Our current model is based on SMT but it would be valuable to explore how the results differ under the assumption of TOFT. The division, mutation, and death of individual cells results in an evolving set of cell populations that can be represented mathematically as a system of differential equations describing a Markov birth-death process. The cellular mutation rate is determined by the amount of DNA damage calculated using the model of selenium metabolism. The output of this model is a hazard function describing the instantaneous rate of cancer incidence as a function of age.

### Trial simulation

We simulated a cancer prevention trial by generating a virtual population of individuals and randomly assigning them to control or supplement groups. Individuals were randomly assigned an age at the start of the trial (mean 62.0 years, standard deviation 2.0 years) and we calculated an age of cancer incidence based on the selenium metabolism and cancer onset models. A large number of trials were simulated with varying values for the parameters but the parameters of the selenium and cancer onset models were the same for all individuals within each trial. Future work may model the variability of individual trial participants using the distributions of the participants of the NPC trial [6], the trial that suggested that dietary selenium supplementation reduced the incidence of prostate cancer. We then used Cox proportional hazard regression on the simulated incidence data to generate hazard ratios and p-values for control versus supplement groups.

These models, when combined, produce a wide range of behaviors that depend on the particular values of the parameters. Many of these parameter values are not precisely quantified. Rather than try to make exact predictions about trial outcomes, we use the models to quantitatively describe the degree to which uncertainty about each parameter value contributes to uncertainty in the trial outcome. We used the trial simulation framework to analyze the selenium model to determine how sensitive simulated trial outcomes were to changes in the model parameters. Our approach was to simulate a large number of trials with varying model parameters. We then used machine learning techniques to analyze the relationship between model parameters and simulated trial outcomes. This allowed us to identify the parameters with the largest influence on trial outcome.

The simulations were run using Matlab and KroneckerBio v0.3.0 alpha, a MATLAB-based toolbox for systems biology model building, simulation and analysis built by members of the Tidor laboratory. Additional file 1: Table S1 lists the lower and upper bound values for the selenium metabolism model parameters and Additional file 1: Table S2 contains the Matlab code used to run the selenium simulations. Additional file 2: Mechanistic selenium metabolism model in SBML format. Nominal rate constant and initial species concentrations are supplied. Generated with COPASI 4.14 (www.copasi.org). Additional file 3: Mechanistic selenium metabolism model in native COPASI file format.

## Results

**Figure 3:**
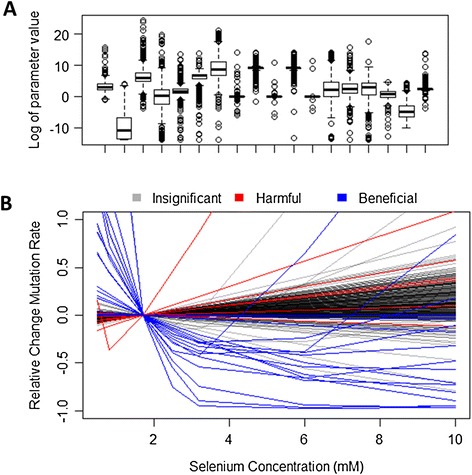
**Model ensemble and simulated outcomes. (A)** Distribution of model parameters for the 502 different selenium models. Horizontal lines and boxes represent the median and interquartile range of the distribution, whiskers show up to 1.5 times the interquartile range, and points represent samples beyond that. Note that parameter values are shown on a natural log scale. **(B)**. Corresponding dose–response curves showing the relationship between the level of selenium and the mutation rate predicted by the selenium model for each set of parameters. All parameter sets are designed to produce the same mutation rate at the baseline level of selenium (1.7 mM). Curves are colored according to the outcome of trials simulated with that parameter set: grey curves correspond to insignificant results, red to harmful results, and blue to beneficial results.

We generated 502 sets of selenium model parameters and simulated several replicated trials for each set. The parameters were constrained to biologically and physically realistic values (e.g., reaction rates must be positive and cannot be larger than diffusion limits; see Additional file 1: Table S1) and consistent with prostate cancer incidence data, but were otherwise randomly distributed. To generate these parameters we first fit the cancer onset model parameters (mutation, death, and birth rates) to Surveillance, Epidemiology, and End Results (SEER) data for prostate cancer incidence.^a^ We then fit the selenium model parameters to produce a mutation rate equal to the fitted cancer onset model mutation rate at baseline selenium levels. The fitted cancer onset model parameters are 3.65 × 10^−5^, 762.2, and 762.0 events per cell per year for mutation rate, birth rate, and death rate, respectively. This corresponds to cell divisions occurring about twice per day and a mutation rate of roughly 10^−7^ per generation, which is consistent with estimates of mutation rates in mammalian cells [20] and birth and death rates in prostate cells [21]. All the fitted selenium models produce the same mutation rate at baseline selenium levels, but even so their individual parameters vary over a very large range (Figure 3A); the constraint of multiple parameter sets that result in the same mutation rate produces correlations among the fit parameters [22]. We found that the average properties of the group of trial simulations converged with just 502 parameter sets, although the parameter space itself was 18-dimensional; the correlations among parameters likely made the effective dimensionality of the space lower.

Each of these models responds differently to increases in selenium from supplementation. The outcomes of simulated trials, in which half of the participants have elevated selenium levels from supplementation, depend strongly on whether the mutation rate decreases, remains relatively constant, or increases with higher selenium levels. Figure 3B shows the mutation rates predicted by all 502 selenium models as a function of selenium level. Mutation rates are expressed relative to the mutation rate at baseline levels of selenium (1.7 mM). Supplementation is treated as raising the selenium level to 2.5 mM and above, for which the different models predict widely varying mutation rates. Curves labeled in blue correspond to models that produced consistent beneficial results in simulated trials, defined as having at least two replicated trials with p-value < 0.05 and a mean hazard ratio < 1.0, while those in red correspond to harmful results, defined as having at least two replicated trials with p-value < 0.05 and a mean hazard ratio > 1.0. Gray curves represent models that did not produce significant harmful or beneficial results.

In general, beneficial trials were projected when the mutation rate decreased from baseline upon selenium supplementation, with variation due to the stochastic nature of the trial simulations. Since mutation rate drives carcinogenesis under the assumptions of somatic mutation theory, this result was expected. What is more elucidating in the model is computed statistical significance of the relationship between selenium supplementation and increased mutation rate. Most models did not produce statistically significant results, with only about 4% of trials (18/502) producing beneficial outcomes (mean hazard ratios ranged from 0.225 to 0.999 with a median of 0.800) and 2% (11/502) producing harmful outcomes (mean hazard ratios ranged from 1.01 to 1.19 with a median of 1.09). For a trial to be judged significant, the result had to be statistically significant to the 95% confidence limit. The observation that only 6% of the trials produced statistically significant results is a reflection of the large uncertainty in the selenium model parameters and how that propagates to produce large uncertainties in changes in mutation rate in response to supplementation. The large uncertainty at the biology level propagates through to a large uncertainty in the trial outcome. This uncertainty could be reduced by conducting experiments to measure or constrain parameter estimates, which may lead to a more focused set of expectations from the trial due to greater certainty of the underlying biology and its connection to disease.

**Figure 4:**
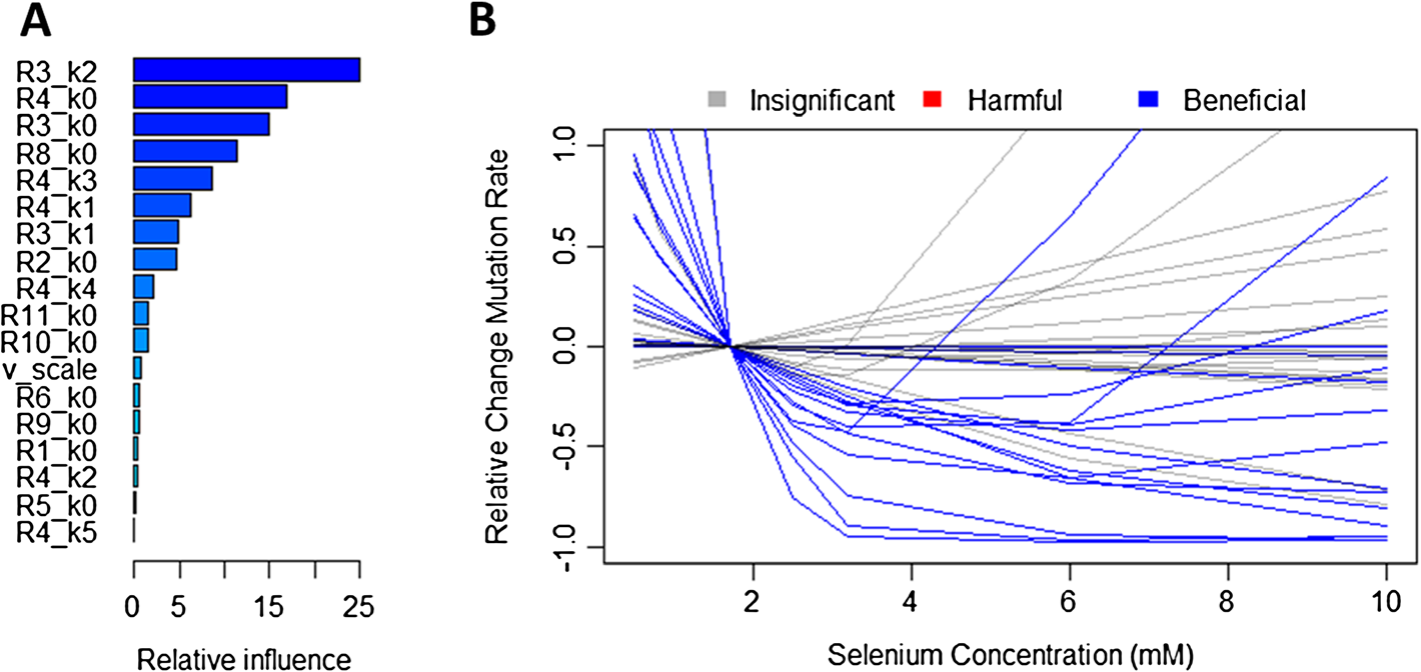
**Identifying features of beneficial trials. (A)**. Relative influence of each selenium model parameter on trial outcomes as identified by the machine learning classifier. Higher relative influence indicates that trial outcomes are more sensitive to variation or uncertainty in the corresponding parameter. **(B)**. Selected curves from Figure 3B for selenium model parameter sets predicted by the classifier to produce beneficial trial outcomes. Not all of these parameter sets produce beneficial outcomes because the classifier is not 100% accurate, but all harmful outcomes are eliminated and the proportion of beneficial outcomes is dramatically increased.

From a VOI perspective, it is helpful to know which measurements should be prioritized to maximize the amount of information gained prospectively about trial outcomes. Our hypothesis is that simulated trials with beneficial results share some common features in the values of their 18 selenium model parameters and thus the biology represented. Since it is difficult to visualize features spanning 18 dimensions, we trained a classification model on the 502 model results to identify the parameters that are most predictive of beneficial results. We split the 502 data points into training (70%) and test (30%) sets and used Gradient Boosted Decision Trees [23,24] to perform the classification. The test set was used to evaluate the performance of the classifier and was not used in training. There were 150 data points in the test set, of which 6 produced beneficial results in trial simulations and 144 produced insignificant or harmful results. The classifier correctly identified 5/6 of these (true positive rate = 83%), while incorrectly classifying 11/144 data sets with harmful or insignificant results as beneficial (false positive rate = 7%). Figure 4A plots the relative importance of each selenium model parameter in determining whether a trial will be beneficial or not. The top four parameters (R3_k2, R4_k0, R3_k0, and R8_k0) are directly related to the rates of reactions producing methylselenol and transporting extracellular selenium into the cell as selenide, as shown in the reaction diagrams in Figure 2A. Because these four reactions are the most predictive of trial outcomes, they are the best targets for conducting further research in advance of a large clinical trial.

These results demonstrate that we can use modeling to identify key areas of uncertainty and suggests strategies to reduce uncertainty in the trial outcome by refining the model parameters and mechanistic understanding. This point is illustrated in Figure 4B, which plots curves for models that the classifier predicts will have a beneficial result, based on the selenium model parameters. Red and gray curves are those that had harmful or insignificant results in trial simulations, while blue curves had beneficial results. A much larger percentage of this restricted set of models produced beneficial results, 41% (15/37) compared to 4% (18/502) with no restriction, and there are no harmful results. In other words, if we knew the selenium metabolism rates for individual trial participants, especially the rates of methylselenol production and selenium transport, we might be much more confident about the probability of obtaining a statistically significant beneficial trial outcome. Furthermore, we can choose to focus on reducing the uncertainty in variables that will lead to the greatest decrease in uncertainty of trial outcome. For example, the most influential variable identified in Figure 4A, R3_k2, varied in value from 10^−6^ to 10^6^, but if we knew that its true value were less than 10, then the percentage of trials that are predicted to be beneficial would increase from 4% (18/502) to 25% (15/61).

## Discussion

VOI provides a formal framework for decision making in the trial planning process. Within this framework, mechanistic modeling would allow researchers to assess available biological knowledge. A systems framework provides a means through which to link data across different levels of modeling in a hierarchical framework.

We suggest that a combination of basic and clinical science, together with the kind of bio-systems modeling presented here, could be used to “pre-test” proposed trial hypotheses, thereby limiting the risk of negative, inconclusive, or adverse trial outcomes. In the VOI framework, this approach can help prioritize further basic biological or preliminary clinical research by highlighting the sensitivity of trial outcomes to uncertainty in specific elements of the underlying biology or the linkages of that biology to phenotype.

We do not advocate creating a single model to predict trial outcomes but instead explicitly modeling uncertainties in the underlying biology and evaluating their impact on trial outcomes. In the case of SELECT, we represented this uncertainty by varying the parameters of the selenium metabolism model. In future models, it would be useful to consider variations in the model structure, such as by incorporating additional metabolic pathways by which selenium and its metabolites can influence cancer onset. Another amplification of the model is modeling past the first cancerous cell to a detectable tumor, including the possibility for immune surveillance to clear the cancerous cell before it becomes a tumor. Here we fit SEER data to parameterize a somatic mutation model of cancer. However, risk and event rates among clinical trial patients can be very different from the general age-matched population represented by SEER, due in part to trial screening criteria. Other models of cancer should be explored, particularly those modeling how mutations are most likely to occur during development compared to tissue maintenance, and that cancer may be due to selection of lineages with pre-existing oncogenic mutations [25]. The connections between levels of the current model hinge on a small number of parameters. Strengthening the robustness of these connections will be important.

The general modeling framework presented here, with components representing molecular events, cancer onset, and disease natural history, is designed to be flexible, allowing models to be added and modified in a modular fashion and developed iteratively. It is currently focused on modeling the biological mechanisms underlying a trial though trials are typically motivated and supported primarily by observational data and previous trial results. Developing integrated and interactive approaches for combining evidence from these two sources is integral for making effective use of all available information in the trial planning process.

## Conclusions

Multiscale modeling as demonstrated herein links the underlying metabolic mechanisms associated with a dietary supplement with the distribution of probable clinical responses to help determine prospectively which model parameters are most influential. This linkage can help prioritize critical information needs during the clinical trial design phase with a goal of reducing uncertainty in probable trial outcomes. Here simulation identified the top four parameters of a published selenium metabolism model in terms of the relative importance to the trial outcomes and linked these parameters to the rates of reaction producing methylselenol and transporting extracellular selenium into the cell as selenide. This demonstrates how identifying the reactions that are most predictive of trial outcomes can help identify targets for research, allowing trial designers to develop information generation strategies to reduce uncertainty in trial outcomes before trials are launched.

## Endnote

^a^Surveillance, Epidemiology, and End Results (SEER) Program (www.seer.cancer.gov) SEER*Stat Database: Incidence - SEER 17 Regs Research Data + Hurricane Katrina Impacted Louisiana Cases, Nov 2010 Sub (2000–2008) < Single Ages to 85+, Katrina/Rita Population Adjustment > − Linked To County Attributes - Total U.S., 1969–2009 Counties, National Cancer Institute, DCCPS, Surveillance Research Program, Cancer Statistics Branch, released April 2011 (updated 10/28/2011), based on the November 2010 submission. Selection criteria: Malignant Behavior, Cases in Research Database, {Race, Sex, Year Dx, Registry, County. Year of diagnosis} = ‘2006-2008’, {Race, Sex, Year Dx, Registry, County.Sex} = ‘Male’, {Site and Morphology. Site rec with Kaposi and mesothelioma} = ‘Prostate’.

## Additional files

Additional file 1: Table S1 Lists the lower and upper bound values for the selenium metabolism model parameters and **Table S2** contains the Matlab code used to run the selenium simulations. Additional file 2
**Mechanistic selenium metabolism model in SBML format.** Nominal rate constant and initial species concentrations are supplied. Generated with COPASI 4.14 (www.copasi.org). Additional file 3
**Mechanistic selenium metabolism model in native COPASI file format.**

## Abbreviations

ODE: Ordinary differential equation
SEER: Surveillance, Epidemiology, and End Results
SELECT: Selenium and Vitamin E Cancer Prevention Trial
SMT: Somatic mutation theory
TOFT: Tissue organization field theory
VOI: Value of information
