# Supplementary material for: Multilevel modeling and value of information in clinical trial decision support
Source: BMC Syst Biol. 2014 Dec 24;8:6. doi: 10.1186/s12918-014-0140-0 (PMC4304628; doi:10.1186/s12918-014-0140-0)
Supplement: Additional file 1: Table S1 — Lists the lower and upper bound values for the selenium metabolism model parameters and Table S2 contains the Matlab code used to run the selenium simulations. [file 12918_2014_140_MOESM1_ESM.docx]

# Additional file 1

Table S1 lists the lower and upper bound values for the selenium metabolism model parameters and Table S2 contains the Matlab code used to run the selenium simulations.

| **Selenium metabolism model parameter name (see Figure 2A for a list of the reactions modeled)** | **Lower Bound** | **Upper Bound** |
| --- | --- | --- |
| v_scale | 1.00E-07 | 1.00E+12 |
| R1_k0 | 1.00E-06 | 1.00E+12 |
| R2_k0 | 1.00E-06 | 1.00E+12 |
| R3_k0 | 1.00E-06 | 1.00E+12 |
| R3_k1 | 1.00E-06 | 1.00E+12 |
| R3_k2 | 1.00E-06 | 1.00E+12 |
| R4_k0 | 1.00E-06 | 1.00E+12 |
| R4_k1 | 1.00E-06 | 1.00E+12 |
| R4_k2 | 1.00E-06 | 1.00E+12 |
| R4_k3 | 1.00E-06 | 1.00E+12 |
| R4_k4 | 1.00E-06 | 1.00E+12 |
| R4_k5 | 1.00E-06 | 1.00E+12 |
| R5_k0 | 1.00E-06 | 1.00E+12 |
| R6_k0 | 1.00E-06 | 1.00E+12 |
| R8_k0 | 1.00E-06 | 1.00E+12 |
| R9_k0 | 1.00E-09 | 1.00E+02 |
| R10_k0 | 1.00E-06 | 1.00E+12 |
| R11_k0 | 1.00E-06 | 1.00E+12 |

Table S1: Selenium metabolism model parameter bounds

| **%** Simulate selenium metabolism model  % Reset random number generation  rng('default');  run_name = 'run';  mutation_rate = 3.65e-5;  mutation_trials = 1;  Se_conc = 1.7e-3; % mM  inner_model_ensemble_size = 500; % number of different selenium metabolism model paramsets for each mutation_rates  %% Simulate selenium metabolism models  % Make output directory  if ~exist(['inner_model_params_' run_name], 'dir')  mkdir(['inner_model_params_' run_name]);  end    parfor i = 1:inner_model_ensemble_size    m = loadModel_inner_massaction;    times = (1:85)';  outputs = ones(size(times));    sd = @(t,yInd,yVal) (1e-2*yVal+1e-6);  measurements = 1e5*mutation_rate*ones(size(times));  obj = constructObjectiveChiSquare_mutationRate_noR7(m, outputs, times, sd, true, measurements);    opts = [];    opts.UseParams = 1:m.nk;  opts.UseICs = [];  opts.UseModelICs = true;  opts.ObjWeights = [1;1];  opts.UseAdjoint = false;  opts.RelTol = 1e-6;    opts.MaxIter = 200;  opts.MaxFunEvals = 10000;    % Original max bounds  opts.LowerBound = [1e-7 1e-6 1e-6 1e-6 1e-6 1e-6 1e-6 1e-6 1e-6 1e-6 1e-6 ...  1e-6 1e-6 1e-6 1e-6 1e-9 1e-6 1e-6]';  opts.UpperBound = [1e12 1e12 1e12 1e12 1e12 1e12 1e12 1e12 1e12 1e12 1e12 ...  1e12 1e12 1e12 1e12 1e2 1e12 1e12]';    opts.Verbose = 2;    tF = 100;  expt = Experiment(m, tF, [], false, false, Se_conc, [], [], [], 'YeastModel');    opts.AbsTol = GoodAbsTol(m, expt, sd, opts);    [mNew,con,G] = FitObjective(m,expt,obj,opts);    parsave(['inner_model_params_' run_name '/inner_fit_' num2str(i) '.mat'],m,mNew,G,mutation_rate)    end |
| --- |
|  |
| function m = loadModel_inner_massaction  m = InitializeModel('CancerModel');  m = AddCompartment(m, 'Solution', 3, 1);  m = AddState(m, 'selenide', 'Solution', 0);  m = AddState(m, 'dmDNA', 'Solution', 0);  m = AddState(m, 'DNA', 'Solution', 25000);  m = AddState(m, 'methylselenol', 'Solution', 0);  m = AddState(m, 'H2O2', 'Solution', 0);  m = AddState(m, 'methylseleninate', 'Solution', 0);  m = AddState(m, 'TrxR', 'Solution', 1e-3);  m = AddState(m, 'TRM', 'Solution', 0);  m = AddState(m, 'E1', 'Solution', 1e-3);  m = AddState(m, 'ESD', 'Solution', 0);  m = AddState(m, 'EML', 'Solution', 0);  m = AddInput(m, 'SeEx', 'Solution', 1, []);  m = AddOutput(m, 'selenide', 'selenide');  m = AddOutput(m, 'dmDNA', 'dmDNA');  m = AddOutput(m, 'DNA', 'DNA');  m = AddOutput(m, 'methylselenol', 'methylselenol');  m = AddOutput(m, 'H2O2', 'H2O2');  m = AddOutput(m, 'methylseleninate', 'methylseleninate');  m = AddOutput(m, 'TrxR', 'TrxR');  m = AddOutput(m, 'TRM', 'TRM');  m = AddOutput(m, 'E1', 'E1');  m = AddOutput(m, 'ESD', 'ESD');  m = AddOutput(m, 'EML', 'EML');  m = AddOutput(m, 'SeEx', 'SeEx');  m = AddParameter(m, 'l', 10*rand);  m = AddParameter(m, 'R1_k0', 0); % set to small  m = AddParameter(m, 'R2_k0', 500);  m = AddParameter(m, 'R3_k0', 10*rand);  m = AddParameter(m, 'R3_k1', 10*rand);  m = AddParameter(m, 'R3_k2', 1e3);  m = AddParameter(m, 'R4_k0', 1e4);  m = AddParameter(m, 'R4_k1', 1);  m = AddParameter(m, 'R4_k2', 1e4);  m = AddParameter(m, 'R4_k3', 1);  m = AddParameter(m, 'R4_k4', 1e4);  m = AddParameter(m, 'R4_k5', 1);  m = AddParameter(m, 'R5_k0', 10*rand);  m = AddParameter(m, 'R6_k0', 10*rand);  m = AddParameter(m, 'R8_k0', 10*rand);  m = AddParameter(m, 'R9_k0', 10*rand);  m = AddParameter(m, 'R10_k0', 1e-3);  m = AddParameter(m, 'R11_k0', 10*rand);  m = AddReaction(m, '', '', 'DNA', 'selenide', 'dmDNA', '', 'R1_k0', '');  m = AddReaction(m, '', '', 'methylselenol', 'H2O2', 'methylseleninate', '', 'R2_k0', '');  m = AddReaction(m, '', '', 'methylseleninate', 'TrxR', 'TRM', '', 'R3_k0', 'R3_k1');  m = AddReaction(m, '', '', 'TRM', '', 'methylselenol', 'TrxR', 'R3_k2', '');  m = AddReaction(m, '', '', 'selenide', 'E1', 'ESD', '', 'R4_k0', 'R4_k1');  m = AddReaction(m, '', '', 'ESD', '', 'EML', '', 'R4_k2', 'R4_k3');  m = AddReaction(m, '', '', 'EML', '', 'E1', 'methylselenol', 'R4_k4', 'R4_k5');  m = AddReaction(m, '', '', 'selenide', '', '', '', 'R5_k0', '');  m = AddReaction(m, '', '', 'methylselenol', '', '', '', 'R6_k0', '');  m = AddReaction(m, '', '', 'SeEx', '', 'selenide', '', 'R8_k0', '');  m = AddReaction(m, '', '', '', '', 'H2O2', '', 'R9_k0', '');  m = AddReaction(m, '', '', 'DNA', 'H2O2', 'dmDNA', '', 'R10_k0', '');  m = AddReaction(m, '', '', 'dmDNA', '', 'DNA', '', 'R11_k0', '');  m = FinalizeModel(m); |

Table S2: Matlab code to run selenium metabolism model using KroneckerBio
